# An Effective Volume Fraction Controls Both Dynamics and Thermodynamics in Vesicle Suspensions with Tunable Electrostatic Interactions

**DOI:** 10.1021/acs.iecr.5c02702

**Published:** 2025-09-12

**Authors:** Annachiara Siciliano, Raffaele Pastore, Francesco Greco, Francesco Rusciano

**Affiliations:** Department of Chemical, Materials and Production Engineering, University of Naples Federico II, Napoli 80125, Italy

## Abstract

Surfactant vesicle suspensions are colloidal systems of great interest in industrial and biomedical applications. In the presence of charged vesicles and electrolytes, electrostatic interactions are crucial to determine their stability and dynamics. To elucidate how volume fraction and electrolyte concentration affect the microscopic structure and dynamics of these systems, here we employ Brownian dynamics simulations of charged spherical vesiscles in sodium-bromide solutions, using interaction parameters measured in previous experiments. Our results identify a colloidal state diagram, where both the dilute-to-dense and the fluid-to-arrested state crossovers shift toward lower volume fractions as electrolyte concentration decreases. We find how interactions augment the first-neighbor distance and shape vesicles’ radial distribution. Based on the microscopic structure, we define an effective volume fraction that collapses onto salt-independent master curves for both dynamic and thermodynamic indicators, effectively making the state diagram one-dimensional. These findings improve the understanding of charged vesicles, opening new ways for predicting and designing the properties of novel formulations.

## Graphical abstract

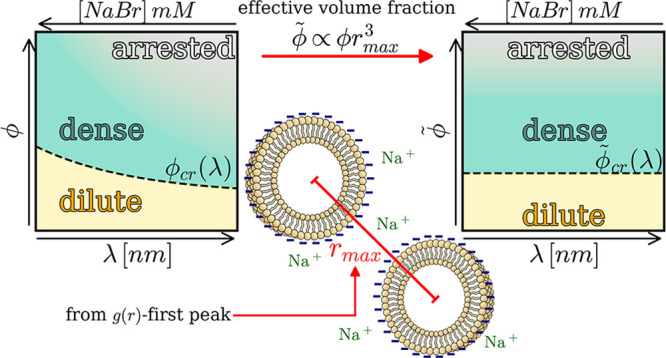

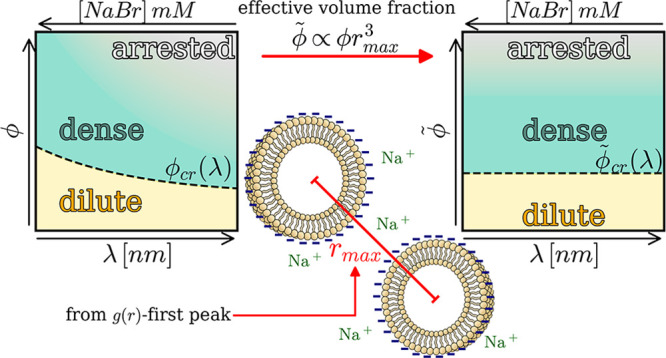

## Introduction

Vesicles are self-assembled closed membranes, consisting of bilayered structures formed by amphiphilic molecules, such as surfactants or phospholipids.^1,2^ The bilayer consists of hydrophilic heads facing the aqueous medium on each side of the bilayer and a corona of hydrophobic tails not in contact with the liquid. These structures play a crucial role in biological processes, including transmembrane transport and encapsulation, and are widely used in industrial and biomedical applications.^3,4^ In biomedicine, vesicles serve as drug delivery carriers because their stability rules encapsulation efficiency^5,6^ and controlled release mechanisms. In industrial formulations, such as detergents and fabric softeners, their phase behavior impacts product stability, overall viscosity, and performances over time.^1,4,7^

From a coarse-grained modeling perspective, vesicle suspensions are colloidal systems where electrostatic interactions significantly affect phase behavior and stability.^8,9^ The celebrated DLVO theory^10,11^ is rather commonly recognized as providing a good starting point for the description of these suspensions.^9^ In the DLVO theory, it is assumed that the interactions between two particles are the superposition of two main contributions: a repulsive one, due to the formation of the so-called Electric Double Layer (EDL) around each particle, and an attractive van der Waals term. Challenging such a classical and simple DLVO two-body picture, many experimental and theoretical works have shown that, under certain conditions, further effective attractive forces may emerge due to electrostatic correlations, ion-mediated interactions, or many-body effects.^12–26^ These interactions can lead to complex phenomenology, including aggregation, phase separation, or structural anomalies. Nonetheless, the simple DLVO framework remains a widely used and practical approach for modeling interactions in like-charged colloidal suspensions, particularly when the electrostatic double layer (EDL) is the dominant contribution.^8^ In charged vesicle suspensions of industrial interest, in fact, the EDL repulsion is often found to be the only relevant interaction, with any attractive contribution being definitively negligible.^8,27,28^

The well-known Yukawa (or screened Coulomb) potential^29^ has been widely employed to model repulsive pair interactions in many kinds of charged systems (e.g., for the so-called dusty, or complex, plasma^30^). In the standard Yukawa potential, the interaction is governed by two independent parameters, namely, the intensity of the potential and the characteristic decay length of the screening effect.^31^ Various numerical works^32–36^ have been devoted to the study of the dynamics and thermodynamics of charged systems, by varying these two parameters, plus temperature, as an external macroscopic control parameter. A Yukawa-like potential is also frequently adopted to model the EDL interaction in colloidal systems.^31,37^ The DLVO theory of colloidal systems gives a physically well-grounded procedure to estimate the values of the pair-interaction parameters, because the potential intensity and the screening length are both determined by the salt content, i.e., by an additional macroscopic control parameter. Of course, the volume fraction of dispersed particles, with finite-size hard cores, also plays a fundamental role in determining the dynamical and structural behavior of colloidal systems.

Earlier works^36,38–41^ have also investigated the interesting possibility of charged systems forming low-density glasses, often with a focus on molecular or point-like systems, and it has been found that electrostatic repulsions shift the glass transition to lower densities, achieving the so-called Wigner glass.^42^ Only more recently, some studies^41,43–46^ have specifically addressed this issue in colloidal (bi- and polydisperse) charged glass-formers, demonstrating that decreasing salt concentration leads to glassy dynamics at significantly lower volume fractions with respect to the pure hard-spheres case. In these systems, dynamic arrest occurs due to a combination of long-range electrostatic repulsion and increasing density, leading to slow relaxation and strong dynamical heterogeneities.

Despite extensive research on charged systems, the structure and thermodynamics of colloidal vesicle suspensions under varying electrostatic conditions are still an open question. Here, we numerically investigate a model of polydisperse surfactant vesicle suspensions in sodium bromide solutions across a broad range of volume fractions and salt concentrations. The parameters of the adopted Yukawa-like potential are taken from a previous independent experimental work,^8^ where they were directly measured for the system at hand (see Materials and Methods section). At variance with the aforementioned works,^43,44^ we will not pursue here the study of glass transition for which the frustration induced by the size disparity among particles is essential; instead, we focus on slightly polydisperse systems spanning different regimes of colloidal particle arrangements, including dilute and dense fluid-like states, and quasi-arrested states.

The results of our Brownian Dynamics simulations point out how salt content influences the “structural crossovers” of the system, i.e., the crossover between the just mentioned colloidal states, and hence allow for the construction of a “colloidal state diagram” of our vesicle system (at room temperature). Examination of the microscopic dynamics then reveals the presence, in the long-term behavior, of a clear-cut scaling law for the diffusivity, which is mirrored also in the constitutive behavior of the suspension, specifically in the appearance of a master curve of the osmotic pressure as a function of an effective volume fraction. Finally, it is found that such an effective volume fraction can be readily interpreted in terms of a microscopic structural feature of the vesicle system.

The article is organized as follows. In the Materials and Methods section, we describe the simulation framework, including system details, interaction potential, and the Brownian dynamics approach used to simulate vesicle suspensions under different electrostatic conditions. In the Results and Discussion section, we first present the “colloidal state diagram”, mapping out the different states observed. Then, we study the diffusion and osmotic properties, and from the emerging scaling laws, we define an effective volume fraction linked to the structural properties of the system. At the end of the section, we discuss at length the novel aspects of our strategy of mapping the system onto a hard-sphere-like one, also in relation to other literature approaches. In the Conclusions, finally, we summarize our findings, emphasizing the impact of electrostatic screening on vesicle suspension behavior. We also discuss potential future directions, including experimental validation and further extensions of our results.

## Materials and Methods

### System Details

We aim to model a Brownian vesicle suspension of industrial interest, namely surfactant di­(alkylisopropylester)-dimethylammonium methylsulfate (DIPEDMAMS) spherical vesicles, in water with added NaBr salt.^8^ Our model consists of *N =* 1500 charged colloidal beads, at a constant temperature *T* = 298*K* and at volume fraction ϕ, defined as the ratio of the total volume occupied by vesicles (calculated from the hard-core diameters σ_*i*_) to the volume of the simulation box *V*_box_: $\phi =\frac{\overset{N}{\underset{i=1}{\sum }}\frac{\pi \sigma_{i}^{3}}{6}}{V_{\mathrm{box}}}≡N\mathrm{v̅}/V_{\mathrm{box}}$ , with *v̅* being the numerical average volume of the particles. The density of the vesicle is taken as that of pure water at the selected temperature, indicating that the total volume occupied by the amphiphilic molecules building up the vesicle membrane is negligible with respect to the volume of the inner part, filled with water. The particle diameters follow a slightly polydisperse size distribution *P*(σ), with a Gaussian profile characterized by a mean diameter σ̅ = 480 nm and a standard deviation δ = 9.6 nm, corresponding to 2% of σ̅. Hence, the diameter distribution *P*(σ) is given by the following probability density:

1 $$P\left(\sigma \right)=\frac{1}{\delta \sqrt{2\pi }}\exp\left[-\frac{1}{2}{\left(\frac{\sigma -\mathrm{\sigma ̅}}{\delta }\right)}^{2}\right]$$

The distribution is truncated, restricting the available diameters to the range σ̅ – 2δ ≤ σ ≤ σ̅ + 2δ in order to exclude extreme values that would have negligible statistical weight (less than 5%), with an unnecessary increase of computational cost.

In charged colloidal systems, the interactions between particles are commonly described using the DLVO theory,^31^ in which the total interaction potential is the sum of an attractive van der Waals term and a repulsive contribution which takes into account electrostatic effects. However, experimental measurements^8^ have shown that the van der Waals attraction is definitively negligible in the DIPEDMAMS vesicle systems that we aim to model. Thus, we have chosen to implement only the screened electrostatic repulsion due to the formation of the electric double layer (EDL) and a hard-core repulsion to prevent nonphysical overlaps between particles^43^ (see Figure 1).

**Figure 1:**
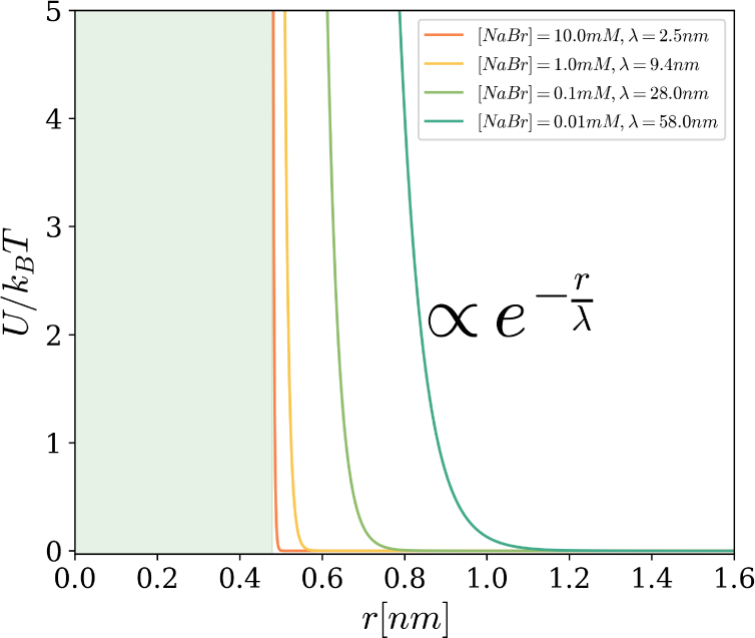
Rescaled pair-interaction potential $\frac{U\left(r\right)}{k_{B}T}$ as a function of center-to-center distance *r* for various salt concentrations (i.e., various Debye screening lengths λ), as reported in the legend. For *r* ≤ σ̅ , the interaction is solely due to hard-core repulsion (green region); for *r* > σ̅, the interaction is modeled via a purely repulsive Yukawa-like potential.

Hard-core repulsion is modeled through an elastic force acting between each pair of overlapping particles, i.e., *F* = −*K*_*n*_Γ, with *K*_*n*_ being an elastic constant and Γ = (σ_*i*_ + σ_*j*_)/2 – |**r**_*i*_ – **r**_*j*_| the overlap between the two particles. The elastic force is activated only if the distance between the centers of mass of two particles *r* = |**r**_*i*_ – **r**_*j*_| is smaller than the sum of their hard-core radii (σ_*i*_ + σ_*j*_)/2, i.e., if Γ > 0. The value of the elastic constant *K*_*n*_ is chosen by imposing the condition that, even for small overlaps (of order 1% of σ̅), the elastic potential energy should be much higher than the thermal energy, i.e., $\frac{1}{2}K_{n}\Gamma^{2}\gg k_{B}T$ , making any overlap practically impossible to occur in fully equilibrated simulations.

The EDL pair interaction potential between particles *i* and *j*, which is activated only when Γ < 0, is given by the Yukawa-like potential:

2 $$W_{\mathrm{EDL}}^{ij}=\frac{\sigma_{i}\sigma_{j}}{\left(\sigma_{i}+\sigma_{j}\right)}\zeta e^{-\frac{r-\left(\sigma_{i}+\sigma_{j}\right)/2}{\lambda }}$$

where

- *r* – (σ_*i*_ + σ_*j*_)/2 represents the distance between the two particles’ surfaces;
- the prefactor ζ determines the magnitude of the electrostatic interaction and is expressed as
3 $$\zeta =64\pi \epsilon_{0}\epsilon {\left(\frac{k_{B}T}{q_{e}}\right)}^{2}\tanh^{2}\left(\frac{q_{e}\psi_{0}}{4k_{B}T}\right)$$
  where ψ_0_ is the surface potential, depending on salt content, ϵ_0_ is the vacuum permittivity, ϵ is the relative permittivity of water, *q*_e_ is the elementary charge;
- λ is the characteristic length-scale of the exponential decay of the Yulawa potential, named *Debye length*, which depends on the ionic strength of the solution.

Notice that the form of the Yukawa potential given in eq 2 is in fact obtained under the well-known Derjaguin approximation,^31^ which is quite common in the context of charged colloids. Such approximation, which effectively integrates out a 1/*r* divergence in the original Yukawa potential (and leads to a purely exponential repulsion), is valid when the screening length is much smaller than the particle radius, which is always the case in our systems.^8^

According to the standard DLVO theory:

4 $$\lambda =\sqrt{\frac{\epsilon_{0}\epsilon k_{B}T}{2N_{A}q_{e}^{2}I_{\mathrm{sol}}}}$$

where *I*_sol_ is the ionic strength of the electrolytic solution, depending on salt content. However, for this study, we did not employ the theoretical formulas: values for λ and ψ_0_ are taken directly from the microscopic measurements of forces between charged DIPEDMAMS bilayers of vesicles in a sodium bromide electrolytic solution.^8,43^

The measured values employed for our Brownian simulations are summarized in Table 1:

**Table 1:** Debye Length *λ* and Surface Potential ψ_0_ Are Given for Different NaBr Concentrations

[NaBr] (mM)	λ (nm)	ψ_0_ (mV)
0.01	58	73
0.1	28	51
1	9.4	21.5
10	2.5	5.6

Experiments in ref. 8 are conducted under infinite dilution conditions, i.e., ϕ → 0. It is expected that, at finite volume fractions, charges on the surface of the particles can contribute additional screening effects, leading to slight modifications in the effective ionic strength of the solvent, which might affect the Debye length λ at low salt concentrations.^43,44^

However, we checked that, in our system, the impact of these corrections on the value of λ due to the crowding of the vesicles is fully negligible. (In the worst investigated case, i.e., the lowest salt concentration of [NaBr] = 0.01 mM and the highest volume fraction ϕ = 0.15 investigated at this salt concentration, the correction is estimated to be below 2%.) Hence, we used the experimental values in Table 1 without applying further corrections. In addition to the system with the above-reported salt concentrations, we also investigated the hard-spheres system, which corresponds to an infinitely high salt concentration and thus an infinite screening, eliminating any electrostatic interaction between the vesicles, i.e., λ = 0.

### Brownian Dynamics Simulations

Brownian Dynamics (BD) simulations were performed in LAMMPS to model the overdamped dynamics of colloidal particles under NVT conditions. The simulations were divided into two parts: a prior equilibration procedure to monitor that all thermodynamic variables attained stationary values; then, a production run during which data were collected for analysis. The particles were randomly distributed inside a cubic simulation box with periodic boundary conditions. The solvent, salty water, was not explicitly modeled, but its effects were simulated by implementing the Langevin thermostat, which maintains a constant temperature of *T* = 298*K* and introduces both random thermal forces and frictional damping on the vesicles due to the solvent. The overdamped Langevin equation governing any particle motion is

5 $$\frac{dr\left(t\right)}{dt}=-\frac{1}{\omega }\nabla U\left(r\right)+\xi \left(t\right)$$

where ω is the friction coefficient, *U*(*r*) is the total interaction potential acting on each particle due to interactions with surrounding particles (within the selected cutoff distance *r*_cut_), and ξ­(*t*) is the random noise due to thermal agitation of the solvent. We have chosen a cutoff distance *r*_cut_ large enough to properly take into account interparticle interactions, i.e., $r_{\mathrm{cut}}=2\sigma_{\max}+O\left(10\lambda \right)$ , where σ_max_ is the maximum diameter in the particle size distribution. Time-integration convergence tests lead us to adopt an optimal integration step d*t* = 1.5*t*_damp_ = 0.02 μs, where *t*_damp_ = *m*/ω is the damping time, and *m* is the vesicle mass (*m* and ω are readily computed from the density and viscosity of water). Values adopted for both parameters, *r*_cut_ and d*t*, provide an optimal balance between computational efficiency and simulation accuracy. The total duration of the production run is 20 s, approximately corresponding to 10^9^ simulated time steps. To compute structural and dynamical properties, all observables were averaged over the ensemble of particles and over different time origins, taking advantage of time-translation invariance at equilibrium. Osmotic pressure has been computed via the formula:

6 $$\Pi =\frac{Nk_{B}T}{V_{\mathrm{box}}}+\frac{1}{3V_{\mathrm{box}}}\overset{N}{\underset{i=1}{\sum }}r_{i}\cdot f_{i}$$

where **r**_*i*_ and **f**_*i*_ are the position and force vectors of particle *i*, respectively. The first term represents the ideal gas contribution, derived from kinetic theory, and the second term is the virial term taking into account particle interactions. A standard formula has been used for the pair correlation function *g*(*r*), thereafter employed to deduce the dilute-to-dense fluid-like crossover in the colloidal state diagram, as discussed in the Results and Discussion section. Specifically, to identify when the colloidal particles are in the dilute/dense state, we analyzed the absence/presence of a minimum in the *g*(*r*) function (within a tolerance of 5%). The appearance of a minimum in *g*(*r*) is universally associated with the onset of some local radial ordering in the system; here, we indeed use this signature to define the crossover from dilute (disordered) to dense (short-range ordered) states.

## Results and Discussion

### Results

As a starting point for the discussion of our results, we display in Figure 2 the colloidal state diagram of our systems at the fixed temperature *T* = 298*K*, as obtained from our BD simulations (symbols) by varying the two macroscopic control parameters, i.e., the volume fraction of the colloidal particles and the salt content in the solvent (water), shown on the upper abscissa. For clarity, on the lower abscissa of the plot, a microscopic description of the system is also given, in terms of the characteristic Debye length λ, specifying the interaction range of the interparticle potential. Data from our simulations of the pure hard-sphere system are also reported in Figure 2, corresponding to the case λ = 0, i.e., the case of a fully screened potential. Background colors in Figure 2 indicate the colloidal states of aggregation of the analyzed systems for any assigned couple (ϕ, [NaBr]) (or (ϕ, λ)) of control parameters. The identification of such “states”, at any given ϕ, was obtained by inspecting structural and dynamical observables^47^ determined in the simulations, as will be detailed below. Here, however, we would like to immediately emphasize that, while the here recognized dilute and dense fluid-like colloidal states are well-defined equilibrium systems, the boundary of the arrested-state region^48^ cannot be sharply defined here, just because a complete dynamical arrest, whether pertaining to a crystalline phase or to a glassy (amorphous) state, was never achieved in our Brownian Dynamics simulations within our simulation time. (In this regard, see the comments following Figure 5.) For this reason, the expected arrested-state region is represented in Figure 2 with continuous shading, to imply that a true arrested state is thought to exist well within that region. A relevant feature of the colloidal state diagram presented here is that upon increasing the salt concentration, the ϕ-span of both fluid states enlarges, eventually reaching the ϕ-windows of the fluid-like pure hard-sphere suspension. Equivalently stated, the dilute-to-dense crossover line ϕ_cr_(*λ*) (defined through the appearance of a minimum in *g*(*r*)-function, as detailed in Materials and Methods) and the fuzzy fluid-arrested state boundary are both shifted toward lower ϕ values by increasing the characteristic length λ of interparticle interaction.

**Figure 2:**
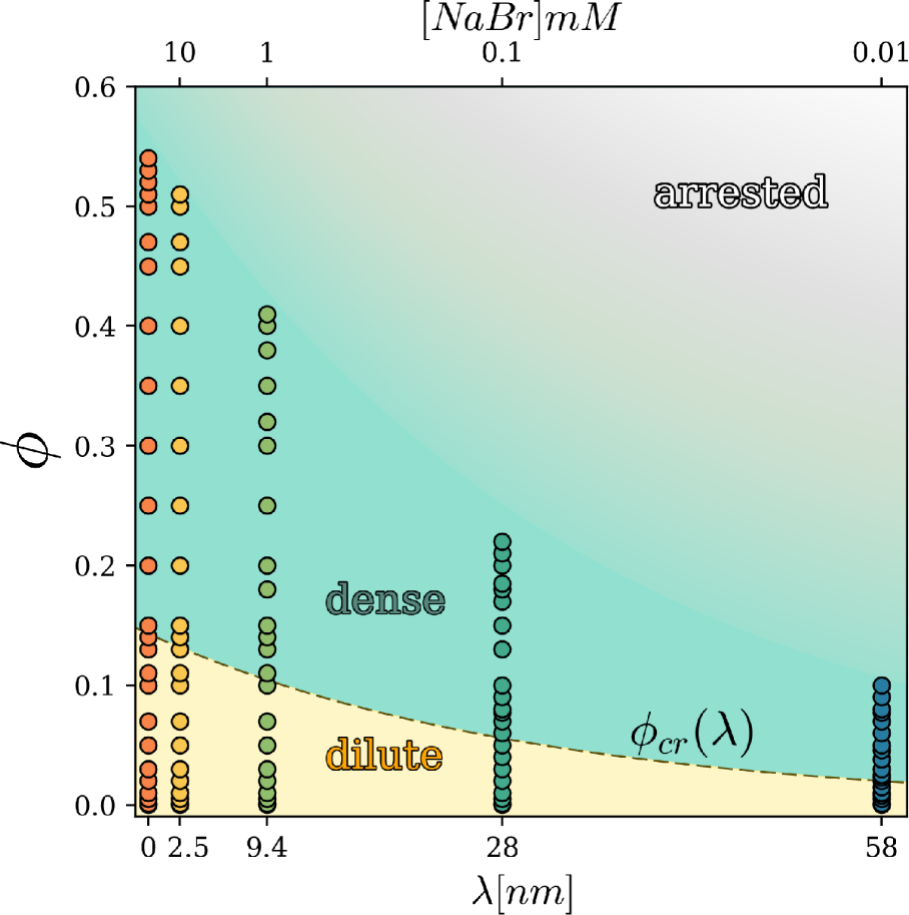
Colloidal state diagram of the charged vesicle system here studied. The vertical axis is the volume fraction, the bottom horizontal axis indicates the Debye length, and the top one shows the corresponding salt concentration. The colored dots represent the simulations performed for various values of ϕ and λ. The dashed line indicates the ϕ_cr_(λ) where the dilute-dense crossover occurs. Note that the upper horizontal scale is provided only as a reference, and its spacing does not correspond to a linear or a logarithmic scale.

Overall, Figure 2 shows that the colloidal system can undergo crossovers to more condensed states either by increasing the volume fraction or by decreasing the salt concentration, i.e., by moving vertically or horizontally, respectively, in the presented colloidal state diagram.

Let us first focus on the changes occurring in the system when we move vertically in the state diagram of Figure 2, i.e., on the effect of increasing the colloidal particles’ volume fraction ϕ at a given salt concentration. Specifically, Figure 3 reports our simulation results in terms of the radial correlation function *g*(*r*; ϕ), i.e., the (static) pair correlation between colloidal particles’ positions, at several ϕ’s, for the largest and the smallest investigated salt concentrations, in panels a and b, respectively.

**Figure 3:**
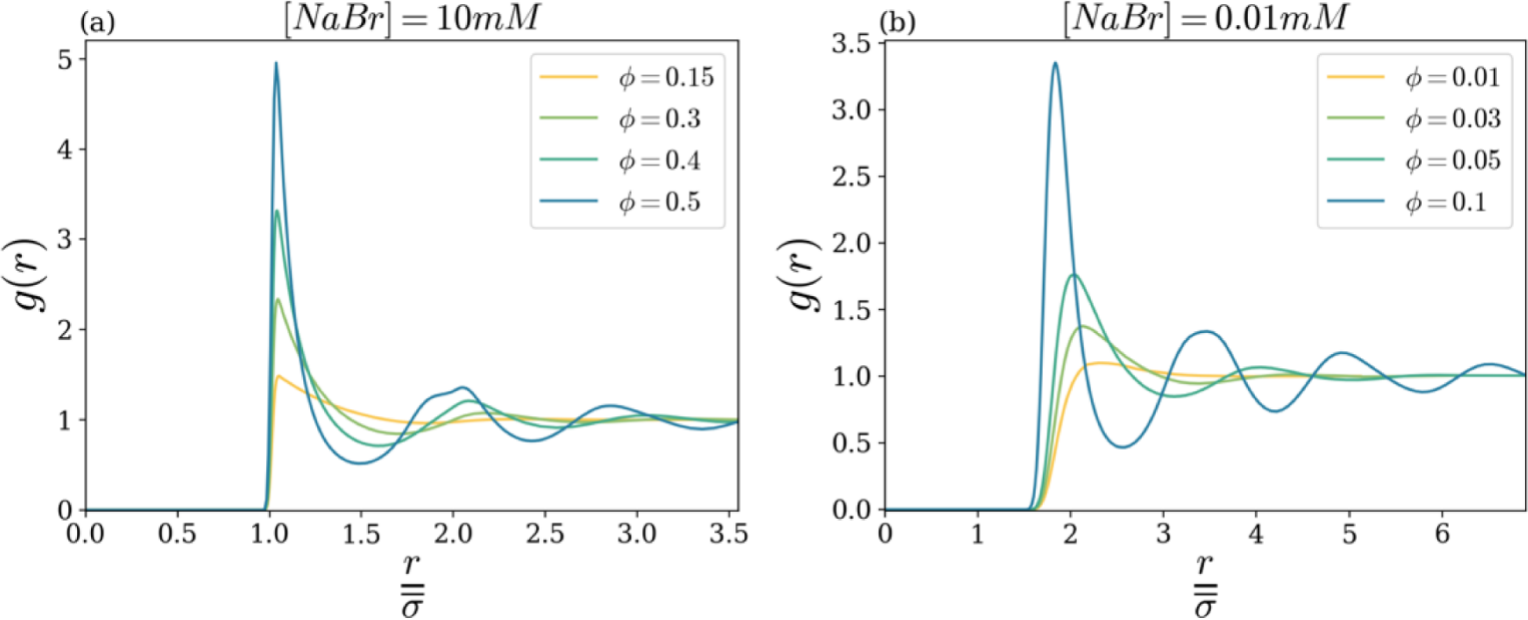
Radial distribution function *g*(*r*) as a function of *r*/σ̅, for different volume fractions ϕ, at fixed salt concentration [NaBr] = 10 mM (a) and [NaBr] = 0.01 mM (b).

At the highest salt concentration [NaBr] = 10 mM (panel a), upon increasing the volume fraction, *g*(*r*) undergoes significant changes. At low concentrations, *g*(*r*) exhibits an almost flat shape, with a single, slightly pronounced peak monotonously decaying to a plateau within a distance on the order of 1 particle diameter. At large ϕ’s, a more complex shape develops, with a large, sharp initial peak followed by several oscillations that decrease in amplitude and eventually fade away at large distances only (several particle diameters). The qualitative change of *g*(*r*) signals the crossover from a dilute-to-dense fluid-like arrangement of the colloidal particles. In this respect, notice also that the appearance of a kind of splitting of the secondary peak at the highest volume fraction can be ascribed to high compactification and incipient solidification of the colloidal fluid. Indeed, the same feature had already been observed in the colloidal hard-sphere system^49^ and supercooled liquids^50^ in proximity to the glassy state.

Similar qualitative changes in *g*(*r*) are apparent also at smaller salt concentrations, as illustrated in Figure 3b for [NaBr] = 0.01 mM. As a matter of fact, for any investigated salt concentration, the dilute-to-dense fluid-like crossover volume fraction ϕ_cr_ was determined by quantitatively identifying the onset of a minimum following the primary maximum of *g*(*r*) (see Materials and Methods for details), in analogy with the gas-to-liquid transition of molecular systems.^51^

By comparing the two panels of Figure 3, three aspects should be stressed: (i) the crossover between dilute and dense fluid-like behaviors is much anticipated (in terms of ϕ) at low salt concentration; (ii) in the condensed state, oscillations of *g*(*r*) switch off at a much larger distance (many particle diameters) in the low salt case; (iii) the position of the main peak markedly shifts to the right with decreasing salt concentrations (it becomes around 2 particle diameters at [NaBr] = 0.01 mM). We notice that the observations reported in (ii) and (iii) qualitatively agree with experimental evidence reported in ref 44.

To further assess the effects of salt concentration on the system structure, we now proceed to inspect the colloidal state diagram by moving along horizontal lines, i.e., by changing the salt density at a fixed particle volume fraction. Figure 4a shows the *g*(*r*) at a volume fraction ϕ = 0.10 for the four salt concentrations inspected. As a reference, we also report in the same panel our BD results for the pure hard sphere system (orange line). It is immediately apparent that the system at the highest salt concentration is essentially indistinguishable from the hard sphere case. As the salt concentration decreases, local inhomogeneities develop: the main peak value grows and shifts its position to larger distances, and secondary oscillations come to the fore. Thus, notwithstanding the (relatively) low value of the particle packing fraction (10%), the colloidal system goes from a dilute-state arrangement to a dense one by reducing the salt content. Of course, the same crossover scenario is present at any other ϕ, here not reported for brevity.

**Figure 4:**
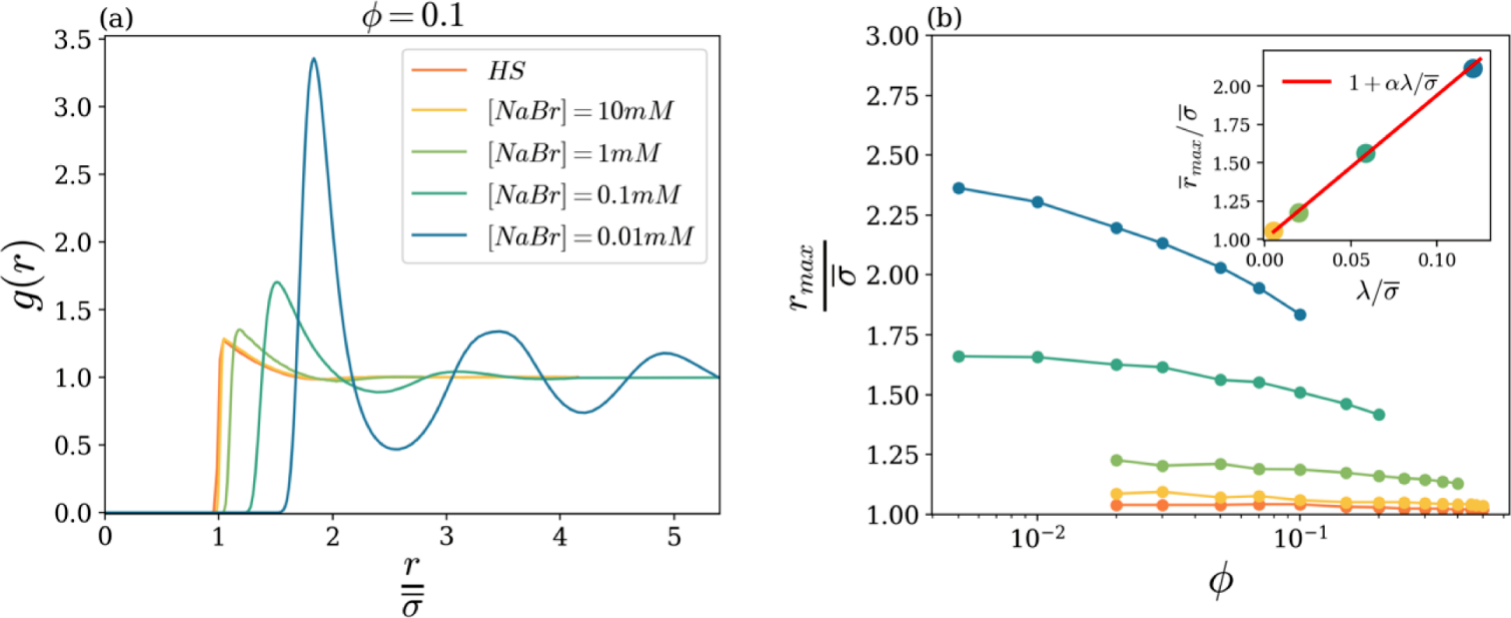
(a) Radial distribution function *g*(*r*) as a function of *r*/σ̅, at fixed volume fraction ϕ = 0.1 and for different salt concentrations, as reported in the legend. (b) Rescaled position *r*_max_/σ̅ of the first peak of *g*(*r*) as a function of ϕ, for different salt concentrations, as shown in panel (a). Inset: rescaled over-ϕ averaged position *r̅*_max_/σ̅ (see text), as a function of λ/σ̅. The red line is a linear fit with α = 9.4.

Panel (b) reports the position *r*_max_(ϕ) of the first peak of *g*(*r*) as a function of ϕ for all of the investigated salt concentrations. For a better comparison with the hard-spheres case, for which the peak $r_{\max}^{\mathrm{HS}}$ is expected to be close to one average particle diameter σ̅, the vertical axis has been rescaled to the value σ̅ = 480 nm. As already observed above, the high-salt situation is really close to that of the hard-spheres system; on decreasing salt concentration, *r*_max_(ϕ) is found to markedly increase with respect to the hard-spheres reference, in agreement with experimental results on charged colloidal suspensions.^44^ Specifically, in the limit ϕ → 0, we find 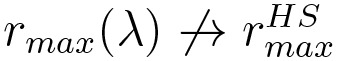 , this difference increasing as salt content is decreased. This early development of structural correlations is a direct signature of the presence of (screened) electrostatic repulsion, which early starts to influence the average interparticle distance. This shows that the Yukawa interaction becomes physically relevant as soon as ϕ is slightly above zero (i.e., in any real physical system).

Microscopically, decreasing the salt concentration corresponds to increasing the Debye length λ. Notice that, in this regard, whatever examined λ value is much smaller than the hard-sphere average diameter, $\mathrm{\sigma ̅}\approx r_{\max}^{\mathrm{HS}}$ , by a factor of around 10–200. In spite of the smallness of the ratio λ/σ̅, we find that the overall increase of *r̅*_max_/σ̅ (*r̅*_max_ being, at each λ, the over-ϕ averaged value) as a function of that ratio is over 100% (see Inset). Indeed, it is

7 $${\mathrm{r̅}}_{\max}=\mathrm{\sigma ̅}+\alpha \lambda$$

with a fitted value of α = 9.4. Consequently, if one were to define an effective volume fraction through the neighbor distance *r*_max_(ϕ; λ), such a volume fraction would be much larger than the actual one, defined through the hard-sphere (mean) diameter σ̅. This point will be addressed in detail in the Discussion subsection.

We go now into the study of the microscopic dynamics of the vesicle suspension. In Figure 5, the particles’ Mean Square Displacement (MSD) is shown as a function of time at a fixed salt concentration by varying the volume fraction (panel a) and at a fixed volume fraction across all salt concentrations (panel b). For reference, the MSD of the hard sphere system is also reported in panel b. Figure 5a shows that, at very low ϕ, MSD is always linear in time, indicating standard diffusive behavior, ⟨*r* ^2^⟩ = 6*D*_0_ *t*, with *D*_0_ representing the (self-)­diffusion coefficient.^52^ By increasing the volume fraction, an intermediate-time subdiffusive behavior ⟨*r* ^2^⟩ ∝ *t* ^α^ (0 < α < 1) arises between the short-time (free) diffusion^52^ and the long-time one, the latter being characterized by lower and lower values of the self-diffusion coefficient *D*(ϕ). The subdiffusive behavior after the initial free motion of any single colloidal particle stems from correlations in the particles’ displacements due to interparticle interactions: as an outcome, the overall dynamics is slowed down in crowded systems. Similar results are found for simulations at different salt contents, which are here not shown for brevity. Figure 5b demonstrates that an analogous slowing down of the dynamics, with the emergence of subdiffusive motion, also occurs by varying the salt concentration, i.e., by increasing the Debye length λ in the interparticle potential, at a fixed volume fraction ϕ. For low λ values, dynamics are essentially indistinguishable from those of the hard sphere system; at the highest investigated Debye lengths, intermediate subdiffusion and the marked decrease of the long-time self-diffusion coefficient are found. Concerning both panels of Figure 5, and the analogous results for all computed MSDs, here not shown, we deem it useful to remind the reader that our simulations are always in an equilibrium fluid state (either dilute or dense) for the colloidal particles. Indeed, the marked slowing down of the dynamics signals that the system is approaching a solid-like state, which was, however, never actually reached in our simulations. As a matter of fact, simulations attempted beyond the highest volume fractions reported in this work could not be fully equilibrated within our available computational time and also showed some solidification nuclei.

**Figure 5:**
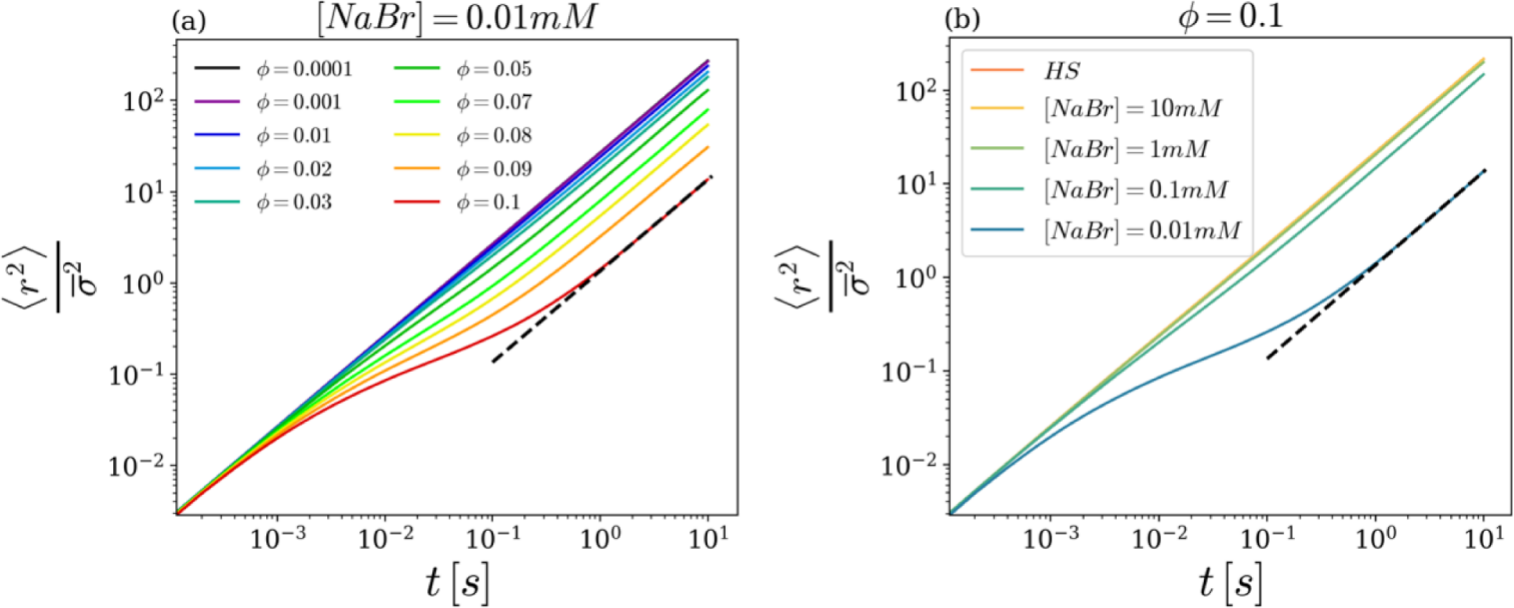
(a) MSD rescaled by squared mean diameter σ̅^2^ as a function of time for different volume fractions at fixed salt concentration [NaBr] = 0.01 mM. (b) MSD rescaled by σ̅^2^ as a function of time for different salt concentrations at fixed volume fraction ϕ = 0.1.

Long-time self-diffusivities at all volume fractions ϕ and at all salt concentrations are reported in Figure 6a, as determined by a linear fit to the long-time data of the MSD (see dashed lines in Figure 5). Figure 6a encompasses the findings of Figure 5, highlighting the slowing of the dynamics that takes place by increasing the volume fraction and/or decreasing the salt content. Note that, whatever the salt concentration, a common plateau is attained in the infinitely dilute condition ϕ→ 0, hence a unique, λ-independent diffusivity, *D*_0_, is found. With respect to the hard spheres case, the decrease of diffusivity with ϕ is markedly anticipated by diminishing the salt concentration, i.e., by increasing the characteristic interaction length λ. In all cases, data indicate a clear-cut tendency toward a vanishing diffusivity, signaling the aforementioned approach to a solid-like state. In view of the qualitatively similar trends observed at different salt concentrations in Figure 6a, it is tempting to check whether a master curve can be obtained by proper shifting along the horizontal axis. This is successfully accomplished in Figure 6b, where we report all data from panel (a) as a function of an *effective volume fraction* ϕ̃, obtained by multiplying the actual volume fraction by a shift factor γ­(λ), only depending on λ:

8 ϕ̃(λ)=γ(λ)ϕ

**Figure 6:**
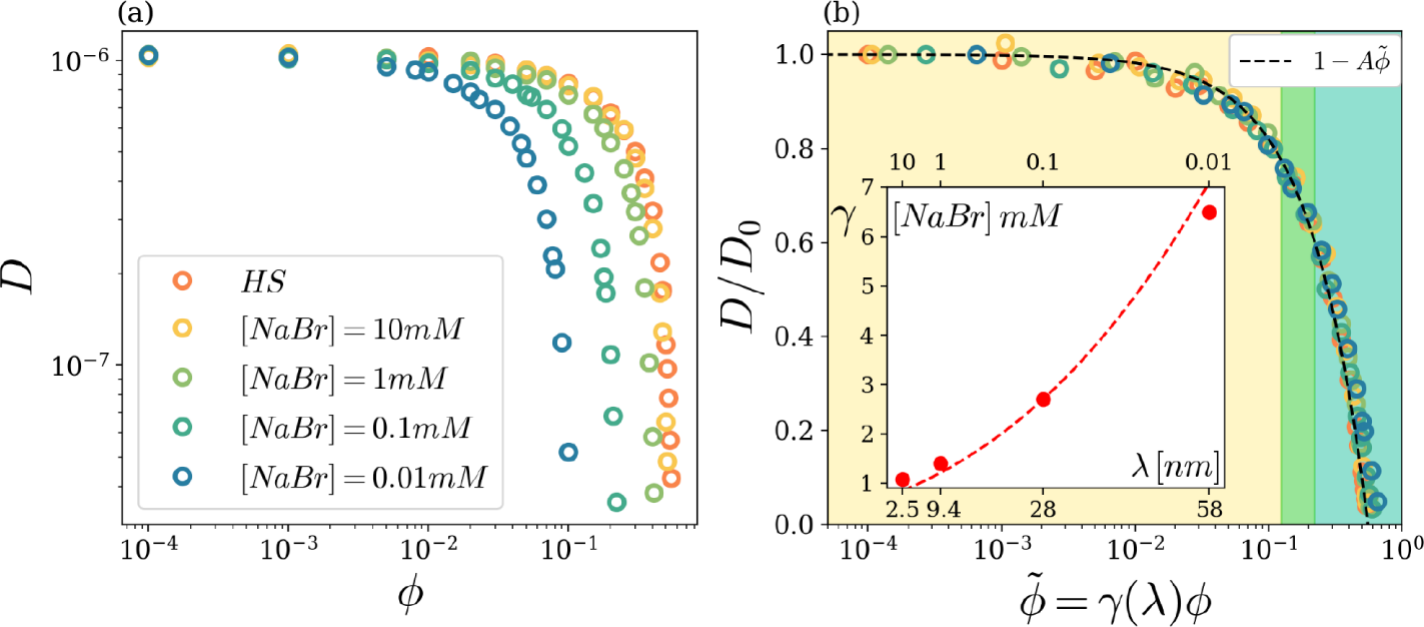
(a) Self-diffusion coefficient *D* as a function of volume fraction ϕ for the salt concentrations reported in the legend. (b) Rescaled diffusion coefficient *D*/*D*_0_ as a function of the effective volume fraction ϕ̃, for the salt concentrations reported in panel (a). The dashed line is a linear fit to the data. Inset: Volume fraction rescaling factor (symbols) as a function of salt concentration. The ded dashed line is the cubic law fit $\gamma ={\left(\chi {\mathrm{r̅}}_{\max}/\mathrm{\sigma ̅}\right)}^{3}$ with χ = 0.9, as described in the text.

The shift factor is reported (symbols) as an inset of panel (b). Notice that, for the largest λ value, the effective volume fraction is almost 7 times larger than the actual one, indicating the huge effect of the EDL interactions on the dynamics: EDL interactions effectively lead to an “expansion” of the particles. Quantitatively, we find that the “expanded diameter” σ̃(λ) of a particle is almost equal to the first neighbor distance, *r̅*_max_(*λ*) (already shown in the inset of Figure 4b). The dashed line in the inset of Figure 6b is indeed given by

9 $$\gamma \left(\lambda \right)={[\mathrm{\sigma ̃}(\lambda )/\mathrm{\sigma ̅}]}^{3}={[\chi {\mathrm{r̅}}_{\max}(\lambda )/\mathrm{\sigma ̅}]}^{3}$$

with a fitted χ value of 0.9. The master curve exhibited by diffusion data in Figure 6b is rather well fitted by the simple functional law *D* = *D*_0_(1 – *A*ϕ̃),^53^ where *A* is a constant parameter. Notice that such a master curve spans throughout the dilute and dense states in the colloidal state diagram of Figure 2. Interestingly, if we focus on the ϕ_cr_(−) crossover line identified in the colloidal state diagram and report in Figure 6b the effective volume fractions corresponding to the actual ones (at each *λ*) for which the crossover occurs, we find that the range of such effective volume fractions is really narrow (a small vertical stripe in panel b). Hence, apart from small unavoidable uncertainties due to the estimates of both the critical ϕ’s and the shifting factors γ’s, the position of the stripe in Figure 6b essentially coincides with the dilute-to-dense crossover. In other words, by looking at Figure 6b, we can state that there is a unique (λ-independent) effective volume fraction ϕ̃_cr_ ≈ 0.15 for the dilute-to-dense crossover. As a relevant consequence, we conclude that in our system, such a crossover occurs at a unique (λ-independent) value of the self-diffusivity, *D* ≈ 0.7*D*_0_.

We now go back to thermodynamic observables and show in Figure 7 the results for the osmotic pressure Π in our colloidal vesicle systems, as directly computed from simulations (see Materials and Methods). Panel (a) reports the osmotic pressure Π, rescaled by the pressure of the “ideal colloidal gas” Π_id_ = *ϕk*BT/*v̅* (where *v̅* is the average particle volume), as a function of ϕ for all investigated salt concentrations. The computed hard-sphere osmotic pressure, also reported as a reference, is in perfect agreement with the well-known Carnahan–Starling Equation of State (EoS).^54^ It so appears that the ϕ-trend of the osmotic pressure by varying the salt content is the same as for hard spheres, with a marked anticipation of deviation from ideal gas behavior as salt concentration decreases. As an example, the osmotic pressure for the system at [NaBr] = 0.01 mM is more than one order of magnitude larger than the pressure of the hard-sphere system at the same volume fraction, ϕ = 0.1. Once again, it is found that the effect of the EDL interactions on the macroscopic observables of our charged vesicle suspensions is a massive one. Once again, as it already occurred for diffusivities (see Figure 6a), the similarity of the osmotic pressure trends with decreasing salt contents, i.e., with increasing Debye length *λ*, calls for an attempt to rescale the horizontal axis in order to check the presence of a master curve. This is accomplished in panel (b), where osmotic pressure datasets have been rescaled by using *the same shifting factors* adopted for the rescaling of diffusivities in Figure 6b, and the results are extremely satisfactory. All systems behave as predicted by the Carnahan–Starling EoS, if effective volume fraction ϕ̃ is properly introduced. In Figure 7b, we have also included the van der Waals EoS (as derived for the hard-sphere case) and the dilute-dense fluide states colored background, as inferred from the colloidal state diagram in Figure 2. Panel (b) shows that the deviations from ideal gas behavior, which are already at play at very small effective volume fractions, are quite well described by the van der Waals law up to ϕ̃ around 0.05–0.1. Rapidly beyond this point, due to increasing interactions among particles, more marked deviations eventually lead the system to a dense state at ϕ̃_cr_ ≈ 0.15, where deviations from the ideal gas pressure are large around 100%.

**Figure 7:**
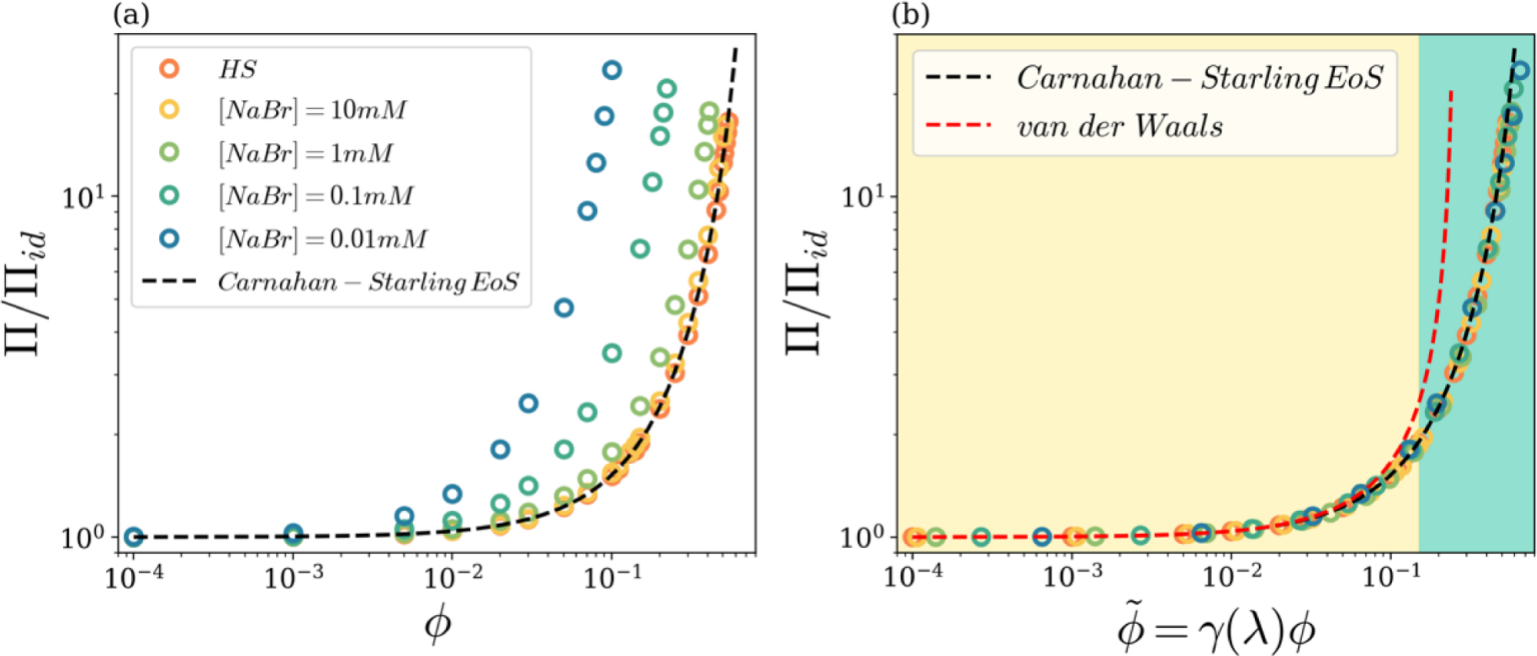
(a) Equilibrium osmotic pressure Π rescaled by the ideal colloidal gas pressure Π_id_, as a function of the volume fraction ϕ, for different salt concentrations, as reported in the legend. (b) Equilibrium osmotic pressure Π rescaled by the ideal colloidal gas pressure Π_id_ as a function of the effective volume fraction ϕ̃, for the same salt concentrations as in panel (a). The dashed lines are the Carnahan–Starling and the van der Waals Equation of State for the hard-sphere system.

Finally, in Figure 8, the colloidal state diagram already presented in Figure 2 is replotted in terms of the newly defined effective volume fraction ϕ̃. As a matter of fact, this figure definitely demonstrates that the state diagram of our charged vesicle system is monodimensional throughout the fluid state, i.e., independent of λ, under the adopted rescaling. All the effects due to the screening of the repulsive intervesicle interaction brought in by the salt contents in the solvent are fully and simply accounted for by the introduction of the effective volume fraction ϕ̃.

**Figure 8:**
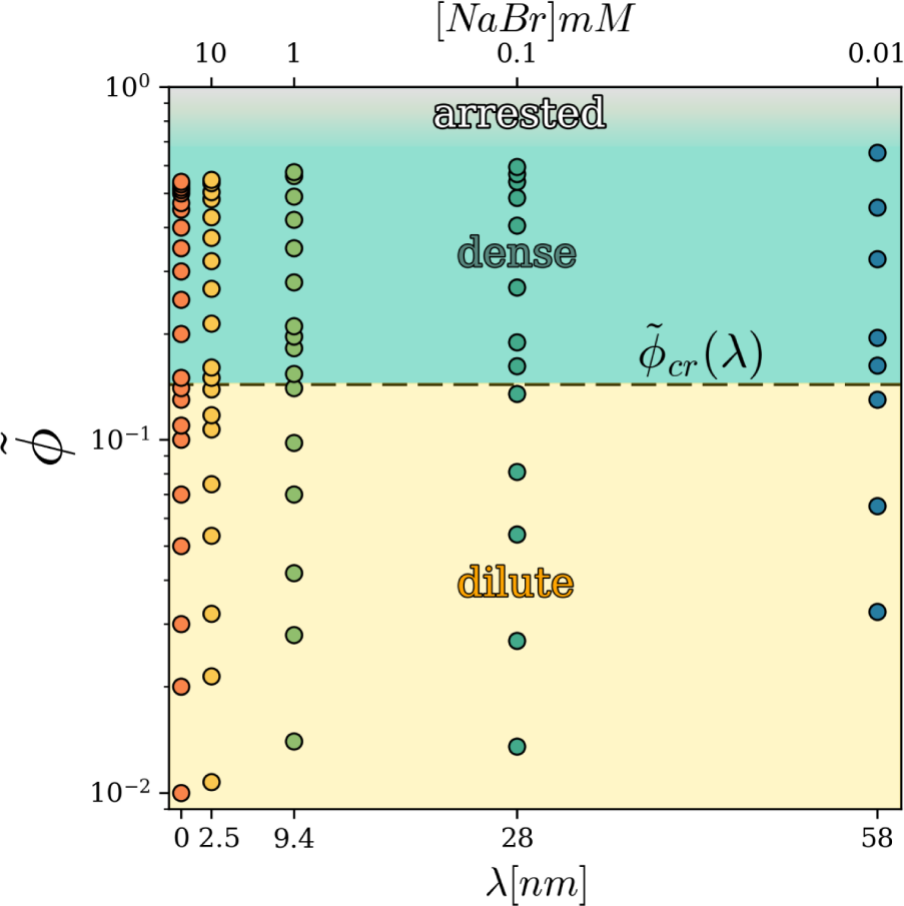
Rescaled colloidal state diagram of the charged vesicle system. The vertical axis is the effective volume fraction, the bottom horizontal axis indicates the Debye length, and the top one shows the corresponding salt concentration. Colored dots represents simulations.

### Discussion

Mapping soft pair interaction potentials onto effective hard-sphere models remains a long-standing and unresolved challenge in soft matter physics.^55,56^ Numerous strategies have been proposed in the literature, yet no universally accepted procedure exists. It is widely acknowledged that different mapping criteria can yield significantly different estimates for the effective hard-sphere diameter or volume fraction.^55^ Most previous studies have focused on identifying effective hard-sphere parameters through theoretical approximations or by collapsing experimental or simulation data, particularly for systems governed by Lennard–Jones, Weeks–Chandler–Andersen, or other soft interaction potentials. However, only a limited number of studies^53,57,58^ have addressed this issue quantitatively in the context of charged colloidal suspensions, where long-range electrostatic interactions complicate the mapping procedure by introducing repulsive forces well beyond the particle contact distance.

In those latter systems, some experimental and numerical studies suggested that the effective hard-sphere diameter σ̃ increases approximately linearly with the Debye screening length λ, following a relation of the form:

10 σ̃≈σ̅+αλ

with α ≈ 1.^44,53,57,59^ In the experimental studies, eq 10 relies on data collapses of a single macroscopic observable (mainly viscosity curves). Our finding in this work is in qualitative agreement with eq 10, with $\mathrm{\sigma ̃}={\left(\frac{6V_{\mathrm{box}}\mathrm{\phi ̃}}{\pi N}\right)}^{1/3}={(\frac{6V_{\mathrm{box}}\gamma \left(\lambda \right)\phi }{\pi N})}^{1/3}=\mathrm{\sigma ̅}\gamma {\left(\lambda \right)}^{1/3}$ and γ­(λ) defined via eq 7 and eq 9. However, our constant α is 1 order of magnitude larger than previously reported values. Several factors may underlie this large discrepancy:

(1) a possibly enhanced influence of EDL interactions in our system as compared to those in refs 53,57,59 , suggesting that the α value could be markedly influenced by thermodynamic or physicochemical parameters such as temperature, absolute particle size, polidispersity, and effective surface charge;
(2) the systems studied in those works might lie in a range of the ϕ−λ parameters space that is closer to the dynamical arrest as compared to ours. In such a case, it is expected that the scaling laws here identified no longer hold uniformly across all observables (e.g., different observables may exhibit different α coefficients): the well-known breakdown of the Stokes–Einstein relation^60–64^ between viscosity and diffusivity, commonly reported in glass-forming systems (including charged colloidal ones^39,43^ ) is a clear indication in this direction;
(3) finally, the proportionality constant α may of course be affected by hydrodynamic interactions, which are neglected in our simulations but are obviously present in experiments. A naively plausible, but absolutely nontrivial explanation, could be that hydrodynamic interactions may result in a partial screening of EDL forces, thus reducing their impact on short-range coordination and leading to smaller α-values. Indeed, the role of hydrodynamic interactions in colloidal systems remains subtle and a topic of ongoing debate. Some theoretical studies have shown that hydrodynamics can introduce tricky effects in dilute charged suspensions, although their quantitative impact on ensemble observables, like diffusivity and viscosity, is generally quite modest.^65–69^ However, incorporating hydrodynamic interactions at high volume fractions remains a challenging issue, both on numerical and theoretical levels. Whether the scaling behavior reported here and its interpretation remain valid in the presence of such effects is an open question that will require dedicated and extensive further investigation.

As a matter of fact, our work provides a systematic investigation of the scaling behavior of different macroscopic observables (diffusivity and osmotic pressure) in terms of an effective volume fraction ϕ̃ (a function of salt concentration). Relying on a robust and consistent definition of ϕ̃, σ̃ is directly connected to a sharply defined microscopic feature of the system: the position *r*_max_ of the *g*(*r*) main peak corresponds to the uniquely defined effective diameter σ̃. Once more, we emphasize that the here obtained radial correlation functions come directly from independent measurements of the interparticle potential^8^ at variance with other studies where no such kind of measurements was performed. In this respect, it seems worth saying that in previous mapping approaches (e.g., by Barker–Henderson and others^55^), ad hoc connections between effective diameter and interparticle potential are adopted, while our volume fraction is directly built on the *g*(*r*), with the potential energy function given once and for all.^8^

In summary, the key results of our study are as follows:

(1) Consistency across macroscopic observables—We demonstrate that the same effective volume fraction ϕ̃ can be independently obtained from both thermodynamic (osmotic pressure) and dynamic (diffusivity) measurements, providing strong internal consistency in the mapping.
(2) Strong deviation from previously proposed scaling law—While our results qualitatively confirm the linear scaling of σ̃ with λ, we find that the proportionality constant α is much larger than previously reported (α ≈ 10), pointing to a much stronger-than-expected influence of electrostatic repulsion on effective packing.
(3) Structural interpretation—We quantitatively connect the effective volume fraction, as determined through data collapse of macroscopic observables, with microscopic structural modifications arising from EDL interactions. In particular, we show that deviations from hard-sphere behavior in *g*(*r*)—notably the expansion of the first coordination shell—fully account for the observed shift in *ϕ̃*, thus offering a direct structural explanation for macroscopic scalings.

Our comprehensive approach provides new insights into the interplay among interparticle interactions, microscopic structure, and macroscopic response in charged vesicle suspensions and suggests a framework for mapping into the effective hard-sphere behavior of other charged colloidal systems.

## Conclusions

In this work, we have studied, by means of Brownian dynamics simulations, a model of slightly polydisperse charged vesicle suspensions in salty water, a system of relevant interest in chemical and biochemical engineering and for industrial applications. The intervesicle interactions are modeled through a Yukawa potential, accounting for EDL repulsions, augmented by a hard sphere contribution; the solvent is implicitly considered in the simulations, with the Debye screening length λ depending on salt concentration. Our investigation focuses on a well-defined set of measured constitutive parameters, which has been directly taken from literature.^8^

Our results demonstrate that such a colloidal system can undergo transitions by varying vesicle volume fraction ϕ and/or salt concentration. From a dilute, fluid-like colloidal state, systems at high volume fractions and/or low salt contents achieve a condensed state. The dilute-to-dense fluid-like crossover line ϕ_cr_(λ) and (possibly) the fluid like-to-arrested state crossovers are anticipated when salt content is decreased. The complete “vesicles state diagram” is obtained by inspecting the qualitative differences in the colloidal arrangement, as measured via the radial distribution function. The corresponding changes in the dynamics are studied through analysis of the particles’ Mean Square Displacement and the ensuing determination of self-diffusion constants. Interestingly, the crossover ϕ_cr_(*λ*) between dilute and dense state behaviors, identified by inspecting changes in structural observables (Figures 3 and 4), is found to be intimately related to the onset of a marked slowing down of the dynamics taking place at the onset of dense, fluid-like arrangement.

From the point of view of the colloidal structure, a remarkable finding of our work is that the value of the screening length strongly affects the position of the first coordination shell; this leads to the microscopic identification of an effective particle diameter σ̃, and, hence, of an effective volume fraction ϕ̃, to take into account interparticle interactions in terms of steric effects.

As a matter of fact, an effective volume fraction has also been separately determined here from the scaling behavior exhibited by either dynamical or thermodynamical macroscopic indicators: diffusivity, as mentioned above, and osmotic pressure, respectively. As a main result of the present work, we find that those different pathways to evaluate the effective volume fraction, i.e., from the microscopic observation of the first-*g*(*r*)-peak shift or from the master scaling of macroscopic observables *D* and Π, are in extremely good agreement: they come up to the same effective volume fraction ϕ̃. As a future perspective, it would be interesting to make a detailed comparison between our *g*(*r*)-based mapping strategy (with its results on the vesicle system) and other existing mappings, mainly based on the determination of a characteristic length from the potential energy function.

It is worth signaling that the here reported trends for the osmotic pressure, as obtained from our simulations, are in qualitative agreement with experimental results on various colloidal charged dispersions with different salts, in particular regarding the tendency to show master curve scalings.^70–72^ In any case, some caution is required when comparing our findings with experiments because of the above-discussed possible effects of hydrodynamic interactions among colloids.^52^

A further delicate issue certainly is the polydispersity of the vesicle suspension, which itself is often poorly characterized experimentally: clarifying the effects of polydispersity both on thermodynamic and dynamical scalings is a difficult task, not yet extensively addressed.

The effective hard-sphere approximation has been proven to be fully valid across a wide range of volume fractions and salt concentrations investigated in our numerical work. However, we expect deviations to occur when the system enters the glass-forming region of the colloidal state diagram, where the structure and dynamics start to uncouple. Such an analysis has not been performed here and will be addressed in the near future.

Finally, we believe that a promising direction for future research on these systems is the investigation of relaxation dynamics and its connection with the overall rheological behavior,^27,73–75^ with particular emphasis on specific industrial applications, e.g., in the processing of liquid fabric softeners or drug carriers.^1,76^ Indeed, the scaling laws and the state diagram identified for the model system studied in this work provide a predictive framework for controlling the state behavior of charged vesicle suspensions by tuning electrostatic interactions and vesicle volume fraction.^77^ This could be particularly helpful for industrial applications,^78^ such as the formulation of stable vesicle-based dispersions in pharmaceuticals and soft-matter products. By adjusting salt concentration and vesicle polydispersity, manufacturers can fine-tune suspension stability, avoiding phase separation or solidification (crystallization/vitrification/gelation),^1,4,79^ while maintaining desired rheological properties. These findings could also guide the design of new self-assembled materials, where phase control is essential for functionality.
